# Outer Membrane Protein 25 of *Brucella* Activates Mitogen-Activated Protein Kinase Signal Pathway in Human Trophoblast Cells

**DOI:** 10.3389/fvets.2017.00197

**Published:** 2017-12-13

**Authors:** Jing Zhang, Yu Zhang, Zhiqiang Li, Jing Liu, Xuehua Shao, Changxin Wu, Yong Wang, Kaisheng Wang, Tiansen Li, Laizhen Liu, Chuangfu Chen, Hui Zhang

**Affiliations:** ^1^College of Animal Science and Technology, Shihezi University, Shihezi, China; ^2^School of Biotechnology and Food, Shangqiu Normal University, Shangqiu, China; ^3^Institute of Fruit Tree Research, Guangdong Academy of Agricultural Sciences, Key Laboratory of South Subtropical Fruit Tree Biology and Genetic Resources Utilization, Ministry of Agriculture, Guangzhou, China

**Keywords:** *Brucella*, 2308ΔOmp25, mitogen-activated protein kinase, cytokines, HPT-8 cells

## Abstract

Outer membrane protein 25 (OMP25), a virulence factor from *Brucella*, plays an important role in maintaining the structural stability of *Brucella*. Mitogen-activated protein kinase (MAPK) signal pathway widely exists in eukaryotic cells. In this study, human trophoblast cell line HPT-8 and BALB/c mice were infected with *Brucella abortus* 2308 strain (S2308) and 2308ΔOmp25 mutant strain. The expression of cytokines and activation of MAPK signal pathway were detected. We found that the expressions of tumor necrosis factor-α, interleukin-1, and interleukin-10 (IL-10) were increased in HPT-8 cells infected with S2308 and 2308ΔOmp25 mutant. S2308 also activated p38 phosphorylation protein, extracellular-regulated protein kinases (ERK), and Jun-N-terminal kinase (JNK) from MAPK signal pathway. 2308ΔOmp25 could not activate p38, ERK, and JNK branches. Immunohistochemistry experiments showed that S2308 was able to activate phosphorylation of p38 and ERK in BABL/c mice. However, 2308ΔOmp25 could weakly activate phosphorylation of p38 and ERK. These results suggest that Omp25 played an important role in the process of *Brucella* activation of the MAPK signal pathway.

## Introduction

*Brucella* spp. are Gram-negative facultative intracellular pathogens that can cause diseases of worldwide significance (1, 2). *Brucella* can cause epididymitis, orchitis, or abortion in animals (3). Infection in humans can cause fever or arthritis (4, 5). It resulted in heavy economic losses (6).

There are three groups of major outer membrane proteins (Omps) in *Brucella* (7). Group 1 Omps consist of two major Omps: Omp10 and Omp19. Group 2 Omps consist of two major Omps: Omp2a and Omp2b. Group 3 Omps consist of two major Omps: outer membrane protein 25 (Omp25) and Omp31. Omp25 was a primary protein that was released by *Brucella* when it invaded host cells (8). Omp25 was involved in attachment or invasion to the host cells and intracellular survival or reproduction of *Brucella*, which plays an important role in *Brucella* virulence. Omp25 mutant was attenuated in animals (9, 10). Therefore, Omp25 is an important virulence factor of *Brucella*.

Mitogen-activated protein kinase (MAPK) is one signal transduction pathway in organisms. It is associated with many profiles and processes of the cell, such as auxesis, development, proliferation, differentiation, and apoptosis (11). MAPK includes four subfamilies: p38, ERK1/2, Jun-N-terminal kinase (JNK), and ERK5 (12). MAPK is implicated in bacterial pathogenesis as demonstrated by the induction of inhibition of ERK1/2 and p38 branches during infection with *Salmonella typhimurium* (13), *Yersinia* (14, 15), *Listeria monocytogens* (16, 17), and *Mycobacterium* (18). In the inflammatory response, MAPK signal pathway can mediate secretion of IL-8, interleukin-10 (IL-10), tumor necrosis factor-α (TNF-α), and other cytokines by epithelial cells (19). In this report, we used S2308 and 2308ΔOmp25 to infect HPT-8 cells and mice, and the expression of cytokines were detected. In addition, we analyzed the effects of Omp25 on the MAPK signal pathway, with the aim to understand the function of Omp25 in the pathogenesis of *Brucella*.

## Materials and Methods

### Ethics Statement

All animal experiments were performed in strict accordance with the Experimental Animal Regulation Ordinances defined by the China National Science and Technology Commission. The study was approved by the Institutional Committee of Post-Graduate Studies and Research at Shihezi University, China (No. 2012-9). Animals are provided with humane care and healthful conditions. All efforts were made to minimize animal suffering.

### Bacterial Strains and Cell Line

*Brucella abortus* 2308 strain was obtained from the Center of Chinese Disease Prevention and Control (Beijing, China). 2308ΔOmp25 mutant was constructed and kept by our laboratory. *Brucella* was cultured in tryptic soy agar or tryptic soy broth (TSB) (Sigma, St. Louis, MO, USA) at 37°C with 5% CO_2_ (vol/vol). The human trophoblast cell line HPT-8 (obtained from Cell Resource Center, IBMS, CAMS/PUMC, Beijing, China) was cultured in Dulbecco’s modified Eagle’s medium (DMEM; Gibco Life Technologies, Rockville, MD, USA) supplemented with 10% fetal bovine serum (FBS; Gibco Life Technologies, Rockville, MD, USA) at 37°C with 5% CO_2_ (vol/vol).

### Mice

Six-week-old BALB/c female mice were obtained from the Experimental Animal Center of the Academy of Military Medical Science (Beijing, China). Animals were maintained in barrier housing with filtered inflow air in a restricted-access room in pathogen-limited conditions. All experimental procedures and animal care were performed in compliance with institutional animal care regulations. And all experimental procedures and animal care were performed in Biosafety Level 3 Laboratory.

### *Brucella* Cell Infection Assay

HPT-8 cells were infected with S2308 and 2308ΔOmp25, as previously described (20). The bacteria for infection studies were prepared before the experiment was executed. S2308 and 2308ΔOmp25 were cultured in TSB at 37°C with 5% CO_2_ (vol/vol) until logarithmic growth phase. Then, 2 × 10^6^ cells/well were cultured in 6-well plates for 24 h at 37°C under 5% CO_2_, and then infected with S2308 or 2308ΔOmp25 at a multiplicity of infection (MOI) of 100 bacteria per cell. Culture plates were centrifuged at 350 × *g* for 5 min at room temperature. At 45 min post-infection, the cells were washed thrice with medium without antibiotics and then incubated with 50 µg/mL of gentamicin (Invitrogen, Carlsbad, CA, USA) for 1 h to kill extracellular bacteria. Afterward, the medium was removed and replaced with fresh DMEM with 10% FBS containing 25 µg/mL gentamicin (defined as time 0). Uninfected cells were used as control.

### Detection of Cytokines

HPT-8 cells were infected with S2308 and 2308ΔOmp25 according to the above description. At 4, 8, 24, and 48 h post-infection, supernatant was collected and filtered with 0.22 µm filter membrane (Millipore, MA, USA). Then, supernatant was centrifuged at 16,000 × *g* for 15 min at 4°C. The levels of TNF-α, interleukin-1 (IL-1), and IL-10 were measured using an ELISA Quantikine Human Kit (R&D Systems, Minneapolis, MN, USA) according to the manufacturer’s instructions. All assays were performed in triplicate and the concentration of each cytokine in the cell supernatant was calculated using a linear regression equation obtained from the absorbance values of standards, according to the manufacturer’s protocol. All assays were performed three times.

### Determination of MAPK Branches Associated with the Secretion of TNF-α

To confirm the MAPK signal pathway associated with the secretion of TNF-α, HPT-8 cells were pre-treated with p38 inhibitor (10 µM), or JNK inhibitor (10 µM) at 37°C for 1 h and then infected with S2308 or 2308ΔOmp25 at a 100:1 MOI according to the above description. At 4, 8, 24, and 48 h post-infection, supernatant was collected and measured the levels of TNF-α according to the above description. All assays were performed three times.

### Western Blotting Analysis

HPT-8 cells were infected with S2308 and 2308ΔOmp25 according to the above description. The activation of p38, ERK1/2, and JNK was detected in infected cells, as previously described (21). Briefly, at 24 h post-infection, supernatant was discarded and cells were lysed in ice-cold Radio Immunoprecipitation Assay Lysis Buffer (Beyotime Institute of Biotechnology, Shanghai, China) for 30 min, then centrifuged at 16,000 × *g* for 30 min at 4°C. The supernatant was collected and concentration was detected with BCA protein assay kit (Sangon Biotech, Shanghai, China). 500 µg protein samples separated by 12% SDS-PAGE and electro-transferred to a nitrocellulose membrane using a Mini Trans-Blot Cell (Bio-Rad, Hercules, CA, USA) at 200 mA for 1 h. Unbound sites on the membrane were blocked in 5% nonfat milk in Tris-buffered saline Tween-20 (TBST) buffer for 1 h at room temperature. Then, the membrane was washed three times with TBST buffer and incubated with rabbit anti-human anti-p38, anti-ERK, or anti-JNK polyclonal antibody (pAb; diluted 1:1,000; Bioworld, Minneapolis, MN, USA) at room temperature for 1 h. After being washed three times, the membrane was incubated with peroxidase conjugated goat anti-rabbit IgG for 1 h at room temperature. After a further washing step, bound conjugate was visualized with an ECL Plus Western Blotting Substrate kit (Thermo Fisher Scientific, USA). All assays were performed three times.

### *Brucella* Infection in Mice and Cytokine Measurement

BALB/c mice were infected with *Brucella* as previously described (22). Briefly, 6-week-old BALB/c female mice (*n* = 5 per group) were randomly divided into three groups. Group 1 and 2 were inoculated intraperitoneally (i.p.) with 200 µL phosphate-buffered saline (PBS; Sigma-Aldrich, MO, USA) containing 1 × 10^6^ CFU of S2308 or 2308ΔOmp25, respectively, group 3 was inoculated i.p. with 200 µL PBS as negative control. Serum samples were obtained from peripheral blood of immunized mice 2, 4, 6, 8, and 10 weeks post-immunization (23). Serum samples were diluted with sample diluent buffer (1:5). The level of TNF-α was measured using an ELISA Quantikine Mouse Kit (R&D Systems, Minneapolis, MN, USA) according to the manufacturer’s instructions. All assays were performed in triplicate and the concentration of each cytokine in the cell supernatant was calculated using a linear regression equation obtained from the absorbance values of standards, according to the manufacturer’s protocol. All assays were performed three times.

### Tissue Specimens

BALB/c mice (*n* = 25 per group) were inoculated with S2308 and 2308ΔOmp25 according to the above description. At 2, 4, 6, 8, and 10 weeks post-immunization, mice were euthanized and uteruses were removed aseptically. The uteruses were collected, formalin-fixed, and paraffin-embedded, as previously described (24).

### Immunohistochemistry

The immunohistochemistry assay was performed as previously described (25). Briefly, the embedded paraffin will be serial sectioned by slicer, with a thickness of 5 µm and mounted on slide glasses coated with poly-l-lysine (Beyotime Institute of Biotechnology, Shanghai, China). Subsequently, the tissue sections were routine deparaffinized and dehydrated with xylene and ethanol. After three times washed with distilled water, the tissue sections were microwaved in 10 mM citrate buffer (pH 6.0; Sigma-Aldrich, MO, USA) at 95°C for 10 min for antigen retrieval and naturally cooled at room temperature. Then, the endogenous peroxidase activity was blocked by incubation with 3% hydrogen peroxide buffer (Sigma-Aldrich, MO, USA) for 10 min at room temperature. The tissue sections were incubated overnight at 4°C with primary antibody (rabbit anti-mouse anti-p38 or anti-ERK pAb; dilution 1:200; Bioworld, Minneapolis, MN, USA). Subsequently, the sections were washed three times with PBS, each time for 5 min. The sections were incubated at 37°C for 30 min with the secondary antibody (biotinylated goat anti-rabbit IgG; dilution 1:500; ZSGB-BIO, Beijing, China) followed by washing three times with PBS, each time for 5 min. Finally, the tissue sections were dropwise added 100 µL DAB Plus Substrate buffer (Thermo Fisher Scientific, USA) containing 2% (v/v) DAB Plus Chromogen (Thermo Fisher Scientific, USA), and the nuclei were counterstained with hematoxylin. The tissue sections were dehydrated, transparented, and sealed with ethanol, xylene, and Neutral gum.

### Statistical Analysis

Cytokine production was expressed as the mean cytokine concentration ± SD. Statistical analysis was performed with Student’s unpaired *t*-test. The differences between groups were analyzed by analysis of variance (ANOVA) followed by Tukey’s honestly significant difference post-test, by comparing all the groups to one another. Results expressed as percentages were analyzed by the Fisher test. The differences between groups were analyzed by ANOVA using SPSS 17.0 software (SPSS, Inc., Chicago, IL, USA). *P* values of <0.05 were considered statistically significant.

## Results

### 2308ΔOmp25 Induces Lower Levels of Cytokines

**Figure 1:**
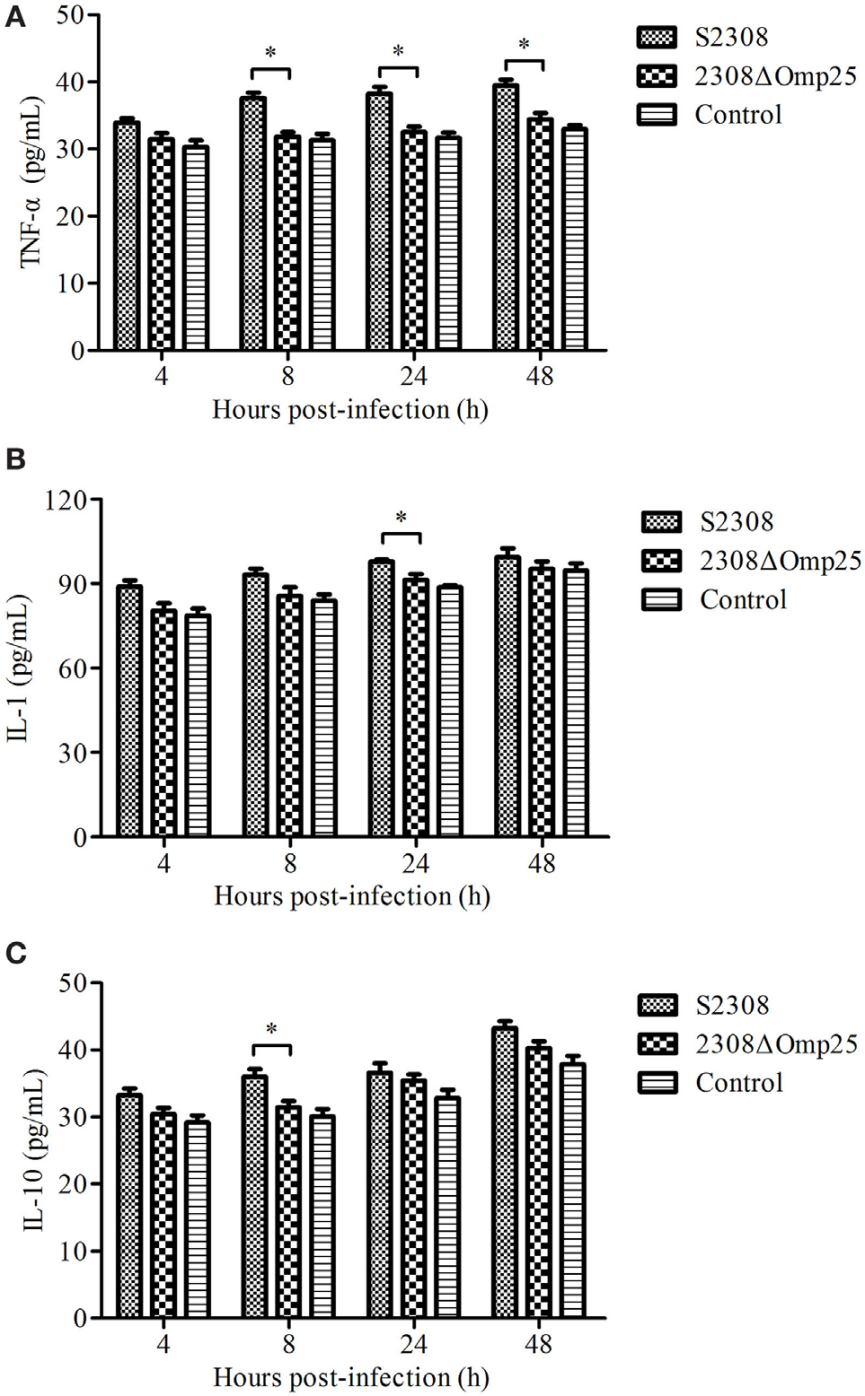
Production of cytokines in S2308 and 2308ΔOmp25-infected HPT-8 cells. HPT-8 cells were infected with S2308 or 2308ΔOmp25 at a 100:1 MOI. Control group was uninfected. At 4, 8, 24, and 48 h post-infection, supernatant samples were collected and TNF-α **(A)**, IL-1 **(B)**, and IL-10 **(C)** levels were assayed by ELISA. Cytokines production is expressed as the mean cytokine concentration ± SD for each group of cells. Significant differences between the S2308 and 2308ΔOmp25 are indicated by * (*P* < 0.05). OMP25, outer membrane protein 25; IL-1, interleukin-1; IL-10, interleukin-10; MOI, multiplicity of infection; TNF-α, tumor necrosis factor-α.

To detect the expression level of cytokines, we collected supernatant from HPT-8 cells infected with S2308 and 2308ΔOmp25 and then monitored expression levels of cytokine TNF-α, IL-1, and IL-10 by ELISA. Supernatant from HPT-8 cells infected with S2308 produced higher amounts of TNF-α (Figure 1A), IL-1 (Figure 1B), and IL-10 (Figure 1C) than did supernatant from uninfected cells (*P* < 0.05) and this difference increased with time. Slightly higher cytokine production levels were observed in 2308ΔOmp25-infected cells than in control cells (Figure 1), but there was no significant difference between 2308ΔOmp25 group and control group (*P* > 0.05).

### Expression of TNF-α Associated with p38 Branch

**Figure 2:**
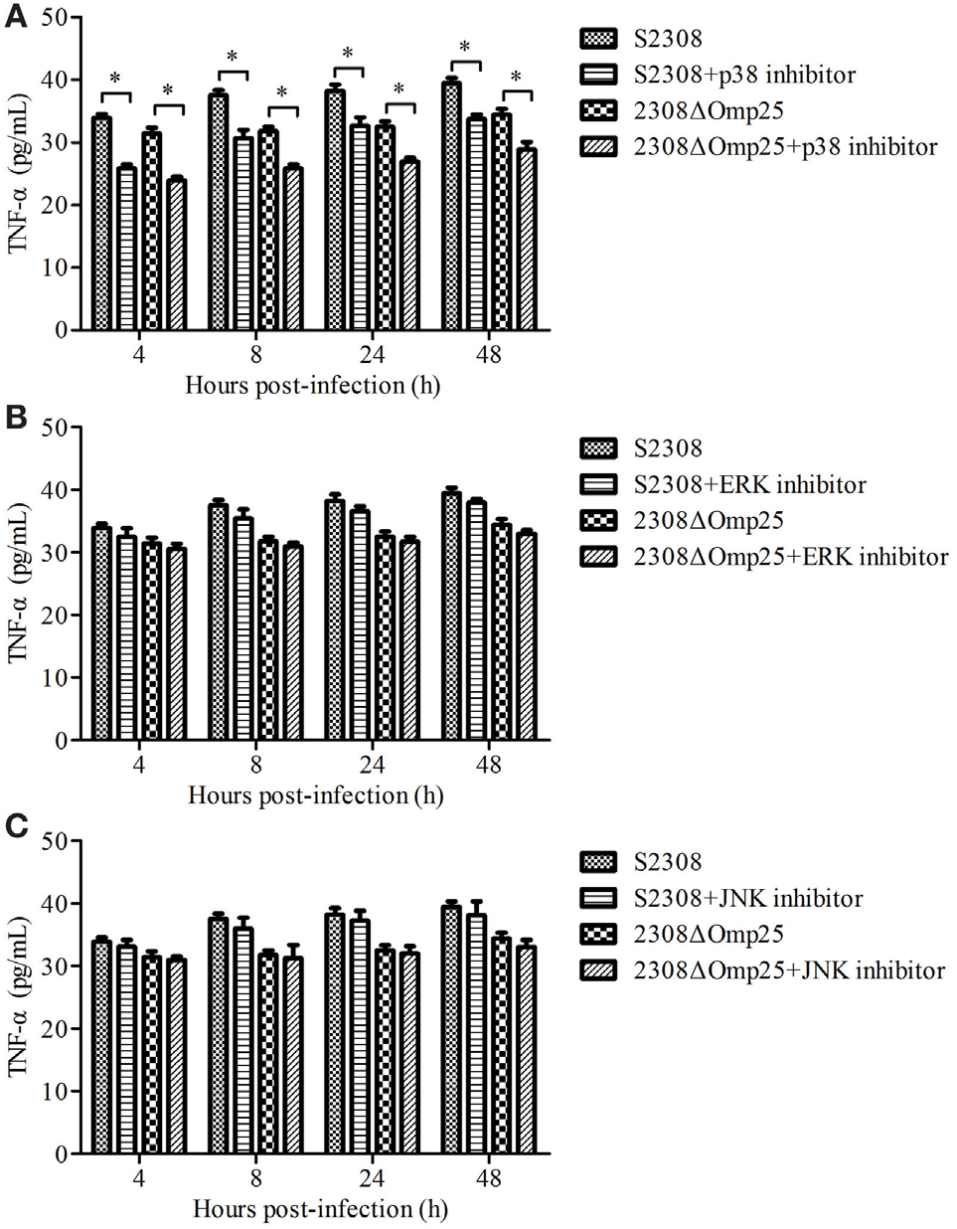
Production of TNF-α in S2308 and 2308ΔOmp25-infected HPT-8 cells. HPT-8 cells were pre-treated with p38 inhibitor (10 µM) **(A)**, ERK inhibitor (10 µM) **(B)**, or JNK inhibitor (10 µM) **(C)** at 37°C for 1 h and then infected with S2308 or 2308ΔOmp25 at a 100:1 MOI. At 4, 8, 24, and 48 h post-infection, supernatant samples were collected and TNF-α levels were assayed by ELISA. TNF-α production is expressed as the mean cytokine concentration ± SD for each group of cells. Significant differences between the S2308 and 2308ΔOmp25 are indicated by * (*P* < 0.05). OMP25, outer membrane protein 25; JNK, Jun-N-terminal kinase; MOI, multiplicity of infection; TNF-α, tumor necrosis factor-α.

We next evaluated which branch associated with secretion of TNF-α. HPT-8 cells were pre-incubated for 1 h with 10 µM p38, 10 µM ERK, or 10 µM JNK inhibitors and then infected with S2308 or 2308ΔOmp25 for 4, 8, 24, and 12 h. The levels of TNF-α were assessed in the supernatants of the S2308 or 2308ΔOmp25-infected p38 inhibitor, ERK inhibitor or JNK inhibitor-treated cells. At 4, 8, 24, and 12 h, the S2308 or 2308ΔOmp25-infected p38 inhibitor-treated cells produced higher levels of TNF-α than S2308 or 2308ΔOmp25-infected cells (*P* < 0.05; Figure 2A). However, there was no significant difference between S2308 or 2308ΔOmp25-infected ERK inhibitor and JNK inhibitor-treated cells and S2308 or 2308ΔOmp25-infected cells (*P* > 0.05; Figures 2B,C). These results showed that p38 branch could induce secretion of TNF-α in S2308 or 2308ΔOmp25-infected cells.

### 2308ΔOmp25 Activates Weak MAPK Pathway

**Figure 3:**
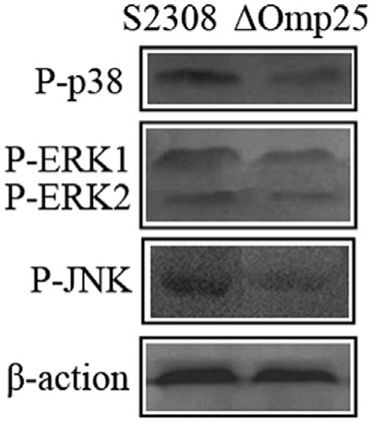
Activation of p38, ERK1/2, and JNK kinases in S2308 and 2308ΔOmp25-infected HPT-8 cells. HPT-8 cells were infected with S2308 or 2308ΔOmp25 at a 100:1 MOI. At 24 h post-infection, supernatant was discarded, and cells were lysed. Then, the activation of p38, ERK1/2, and JNK kinases was detected by Western blotting. Infections with 2308ΔOmp25 induced a markedly weaker stimulation of p38, ERK1/2, and JNK kinases, with S2308 demonstrating a slightly higher capacity of activation than 2308ΔOmp25. OMP25, outer membrane protein 25; JNK, Jun-N-terminal kinase; MOI, multiplicity of infection.

To assess activation of p38 and ERK1/2 kinases, HPT-8 cells were infected with the 2308ΔOmp25 mutant and the parental strain S2308 at a MOI of 100. We found that at 24 h post-infection, the activation process triggered by S2308 resulted in a phosphorylation of the p38, ERK1/2, and JNK kinases (Figure 3). Infections with 2308ΔOmp25 induced a markedly weaker stimulation of p38, ERK1/2, and JNK kinases, with S2308 demonstrating a slightly higher capacity of activation than 2308ΔOmp25 mutant (Figure 3). These results show that OMP25 involved in activating of MAPK pathway.

### 2308ΔOmp25 Induces Lower Levels of TNF-α in Peripheral Blood of Mice

**Figure 4:**
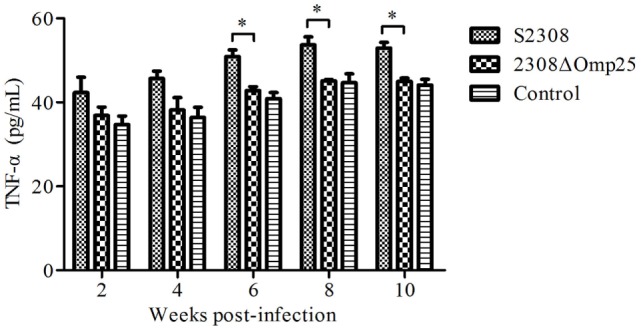
Production of TNF-α in S2308 and 2308ΔOmp25 vaccinated BALB/c mice. BALB/c mice were inoculated with 1 × 10^6^ CFU of S2308 or 2308ΔOmp25. Control groups received PBS. At 2, 4, 6, 8, and 10 weeks post-immunization, serum samples were collected (*n* = 5 per time point) and TNF-α levels were assayed by ELISA. TNF-α production is expressed as the mean cytokine concentration ± SD for each group of mice. Significant differences between the S2308 and 2308ΔOmp25 are indicated by * (*P* < 0.05). OMP25, outer membrane protein 25; TNF-α, tumor necrosis factor-α; PBS, phosphate-buffered saline.

To detect the expression level of TNF-α in animal, we collected sera from mice inoculated with S2308, 2308ΔOmp25, or PBS and then measured expression levels of TNF-α by ELISA. Serum samples from mice inoculated with S2308 produced higher amounts of TNF-α (Figure 4) than did serum samples from mice inoculated with 2308ΔOmp25 or PBS (*P* < 0.05) and this difference increased with time. Slightly higher cytokine production levels were observed in 2308ΔOmp25 immunized mice than in PBS immunized mice (*P* > 0.05) (Figure 4). Total TNF-α levels increased with time.

### Immunohistochemical Staining

**Figure 5:**
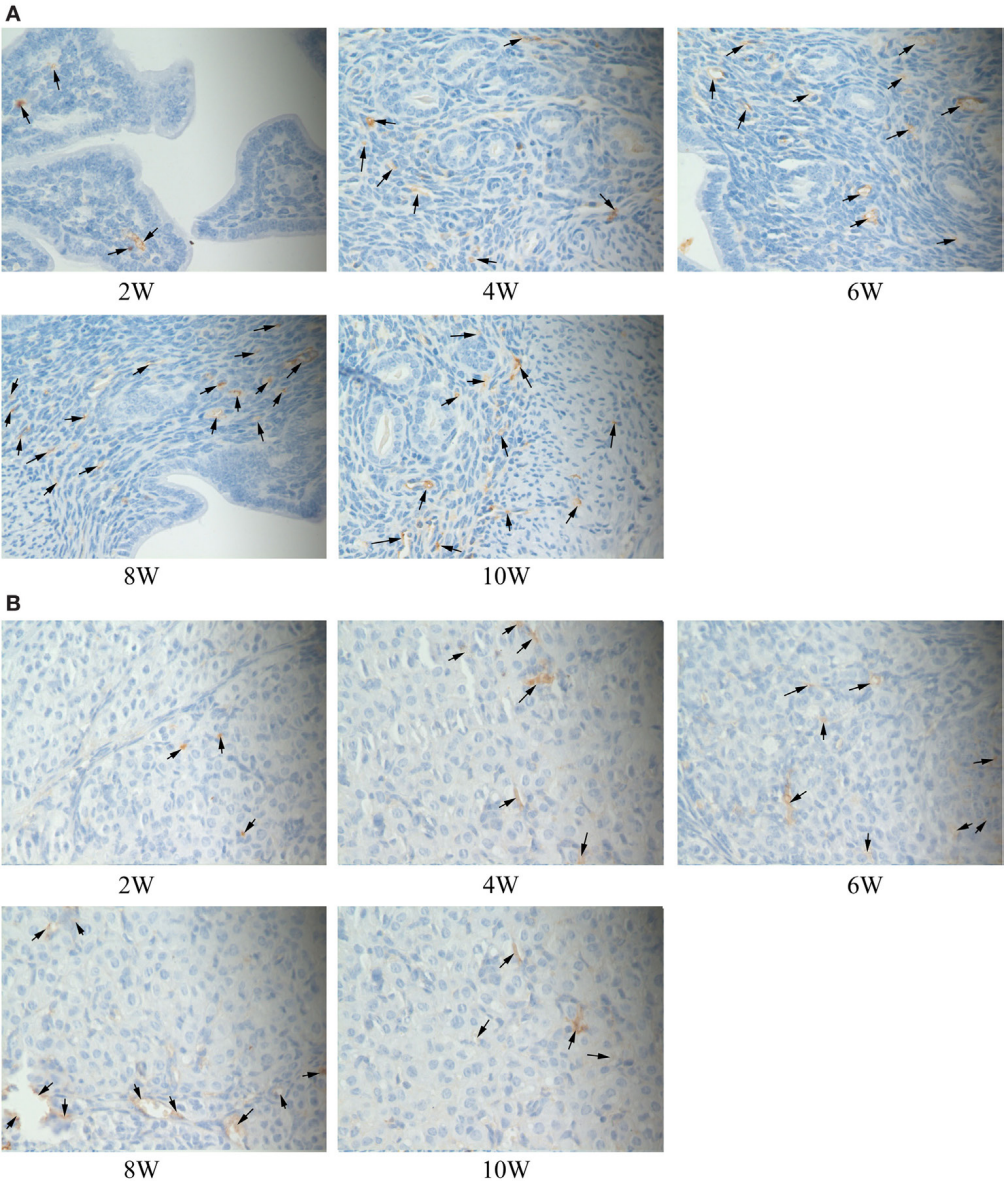
Immunohistochemical detection of phosphorylation proteins in p38 signal pathway branch. BALB/c mice were inoculated with 1 × 10^6^ CFU of S2308 or 2308ΔOmp25. At 2, 4, 6, 8, and 10 weeks post-immunization, uterus tissues were removed and detected by immunohistochemistry. The phosphorylation proteins in p38 signal pathway had been detected and found in the uterus tissues of S2308 immunized mice **(A)**. But the immune complexes of phosphorylation proteins in p38 signal pathway were weakly stained in the uterus tissues of 2308ΔOmp25 immunized mice **(B)**. OMP25, outer membrane protein 25.

**Figure 6:**
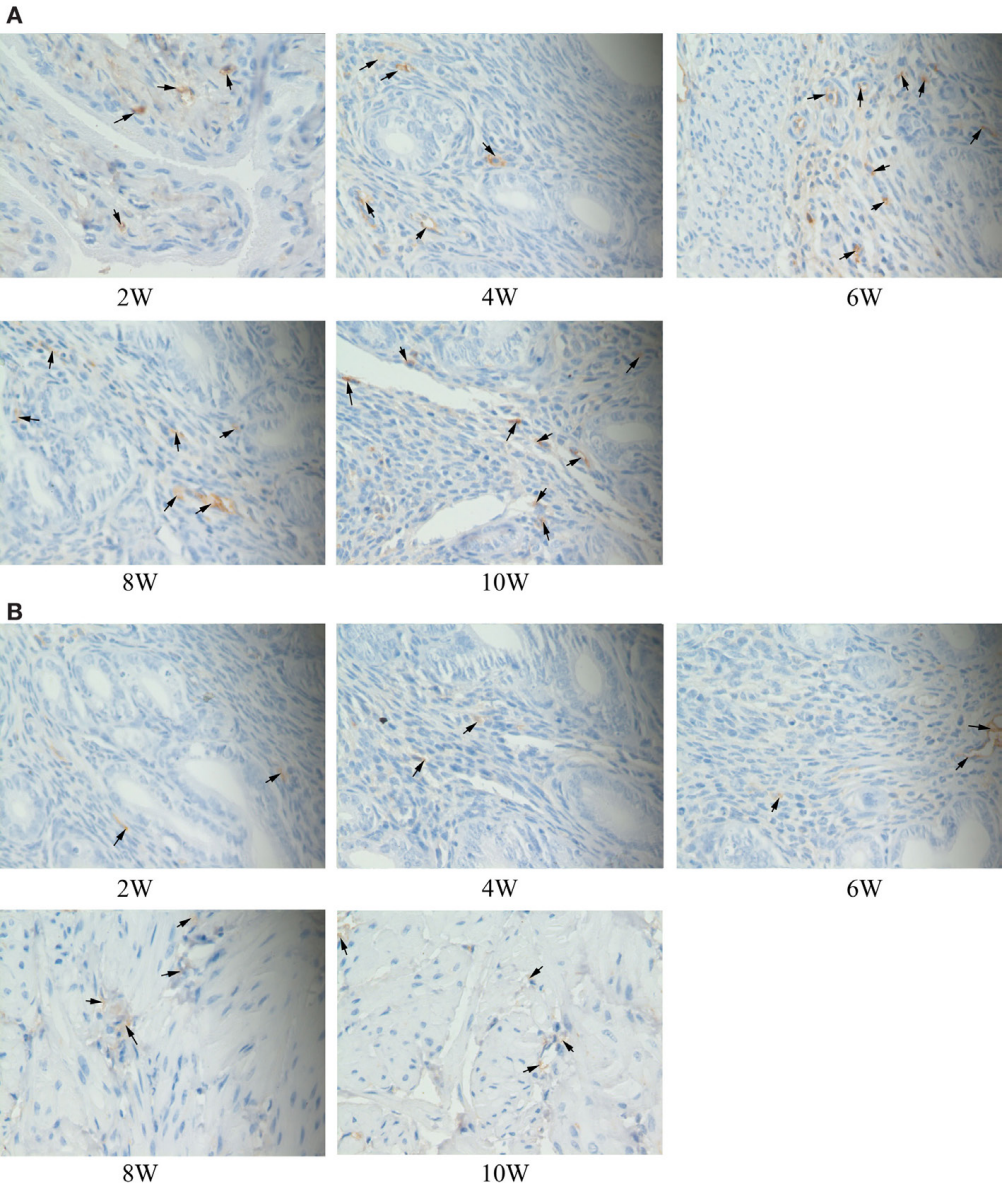
Immunohistochemical detection of phosphorylation proteins in ERK signal pathway branch. BALB/c mice were inoculated with 1 × 10^6^ CFU of S2308 or 2308ΔOmp25. At 2, 4, 6, 8, and 10 weeks post-immunization, uterus tissues were removed and detected by immunohistochemistry. The phosphorylation proteins in ERK signal pathway had been detected and found in the uterus tissues of S2308 immunized mice **(A)**. But the immune complexes of phosphorylation proteins in ERK signal pathway were weakly stained in the uterus tissues of 2308ΔOmp25 immunized mice **(B)**. OMP25, outer membrane protein 25.

The immune complexes of p38 and ERK phosphorylation proteins were located in the cytoplasm of the uterus tissues, and they were strongly stained as tan or brownish yellow. The phosphorylation proteins in p38 and ERK signal pathways had been detected and found in the uterus tissues of S2308 immunized mice (Figures 5A and 6A). But the immune complexes of phosphorylation proteins in p38 and ERK signal pathways were weakly stained in the uterus tissues of 2308ΔOmp25 immunized mice (Figures 5B and 6B). These results showed that S2308 was able to activate phosphorylation of p38 and ERK in BABL/c mice. However, 2308ΔOmp25 could weakly activate phosphorylation of p38 and ERK.

## Discussion

*Brucella* could infect many kinds of cells, but the main host cells are macrophages and trophoblasts (26). In animals, abortion is associated with a rapid proliferation of *Brucella* within the placenta. Trophoblasts are primary cellular targets for *Brucella* in the natural host. The presence of high bacterial loads within placental trophoblasts ultimately results in disruption of the placenta and infection of the fetus. Omps of *Brucella* play an important role in the process of pathogen (27). Omp25 an important Omp, it involved in growth, colonization, and proliferation of *Brucella* (28). The Omp25 mutant strain attenuated *Brucella* infection abilities and changed the response of host cells (10). TNF-α is one of important factors that involved in many of the body’s immune and inflammatory responses (29). The expression of TNF-α was related with Omp25 and ERK pathway (27). Our results found that there was a significant difference in the expression of TNF-α between S2308 and 2308ΔOmp25. These results suggested that Omp25 may play an important role in the progress of expression of TNF-α when *Brucella* infected cells.

Phosphorylation of MAPK p38 pathway acts as a “switch” role in regulating the production of cytokines. The whole process is through a typical pathway: MAPKKK → MAPKK → MAPK (12). SB208035 is the inhibitor of p38 signal pathway. It has been reported that p38 inhibitors could inhibit the production of IL-1, IL-10, and TNF-α in peripheral blood cells (30). TNF-α could activate p38 pathway (31). Our results showed that 2308ΔOmp25 was weaker to activate p38 pathway in MAPK signal pathway than S2308. The reason may be the low expression of TNF-α in 2308ΔOmp25. It suggested that the production of TNF-α may be related with the activation of p38 pathway. When we used p38 inhibitors to deal with HPT-8 cells, and detected the expression of TNF-α in the culture medium. We found that the expression of TNF-α has been inhibited. It suggested that when S2308 or 2308ΔOmp25-infected HPT-8 cells, the p38 pathway in the MAPK signal pathway was related with the expression of TNF-α.

*Brucella* can lead many organs happening pathological damages, particularly in chronic infection stage. In the experiment, we found that the expressions of TNF-α significantly increased when S2308 or 2308ΔOmp25-infected mice or HPT-8 cells. It showed that TNF-α was one of cytokines that happened significantly change when *Brucella* infected hosts (organisms and cells), and it may be related to the progress of inflammation. From the result of immunohistochemistry, we found that phosphorylation proteins of p38 and ERK signal pathway in uterine tissues of mice. In addition, we also found that the immune complexes of phosphorylation proteins in p38 and ERK signal pathways were weakly stained in the uterus tissues of 2308ΔOmp25 immunized mice. These results suggested that Omp25 participated in phosphorylation of p38 and ERK signal pathway proteins. Previous studies have reported that the MAPKs are a target for immune intervention by virulent smooth *Brucella* (32). Our results further suggest that Omp25 played an important role in activating MAPK signal pathway in smooth *Brucella*.

In conclusion, we found that *Brucella* can affect the expression of TNF-α by activating MAPK signal pathway, and the expression was higher in S2308 than 2308ΔOmp25. It suggested that Omp25 played an important role in activating MAPK signal pathway when *Brucella* infected hosts. These results established theoretical foundation for further studying pathogenic mechanisms and proinflammatory mechanisms of *Brucella*.

## Ethics Statement

The study was approved by the Institutional Committee of Post-Graduate Studies and Research at Shihezi University, China (No. 2012-9). All efforts were made to minimize animal suffering.

## Author Contributions

JZ, YZ, LL, and HZ designed the experiments. ZL, JL, YW, TL, and LL performed the experiments and analyzed the data. XS, KW, CW, LL, CC, and HZ contributed reagents/materials/analysis tools. JZ, YZ, ZL, LL, and HZ wrote and revised the paper.

## Conflict of Interest Statement

The authors declare that the research was conducted in the absence of any commercial or financial relationships that could be construed as a potential conflict of interest.
